# Advances of Long Non-Coding RNAs as Potential Biomarkers for Tuberculosis: New Hope for Diagnosis?

**DOI:** 10.3390/pharmaceutics15082096

**Published:** 2023-08-07

**Authors:** Jiaojiao Xia, Yilin Liu, Yuhe Ma, Fen Yang, Yongdui Ruan, Jun-Fa Xu, Jiang Pi

**Affiliations:** 1 Guangdong Provincial Key Laboratory of Medical Molecular Diagnostics, The First Dongguan Affiliated Hospital, Guangdong Medical University, Dongguan 523808, China; 18214682412@163.com (J.X.); liyil6101015@163.com (Y.L.); 15625857518@163.com (Y.M.); yangfen417@163.com (F.Y.); 13829202566@139.com (Y.R.); 2 Department of Biochemistry and Molecular Biology, School of Basic Medical Sciences, Kunming Medical University, Kunming 650500, China; 3 Institute of Laboratory Medicine, School of Medical Technology, Guangdong Medical University, Dongguan 523808, China; 4 The Marine Biomedical Research Institute, Guangdong Medical University, Zhanjiang 524023, China

**Keywords:** long non-coding RNA, tuberculosis, function, biomarker, diagnosis

## Abstract

Tuberculosis (TB), one of the top ten causes of death globally induced by the infection of Mycobacterium tuberculosis (Mtb), remains a grave public health issue worldwide. With almost one-third of the world’s population getting infected by Mtb, between 5% and 10% of these infected individuals are predicted to develop active TB disease, which would not only result in severe tissue damage and necrosis, but also pose serious threats to human life. However, the exact molecular mechanisms underlying the pathogenesis and immunology of TB remain unclear, which significantly restricts the effective control of TB epidemics. Despite significant advances in current detection technologies and treatments for TB, there are still no appropriate solutions that are suitable for simultaneous, early, rapid, and accurate screening of TB. Various cellular events can perturb the development and progression of TB, which are always associated with several specific molecular signaling events controlled by dysregulated gene expression patterns. Long non-coding RNAs (lncRNAs), a kind of non-coding RNA (ncRNA) with a transcript of more than 200 nucleotides in length in eukaryotic cells, have been found to regulate the expression of protein-coding genes that are involved in some critical signaling events, such as inflammatory, pathological, and immunological responses. Increasing evidence has claimed that lncRNAs might directly influence the susceptibility to TB, as well as the development and progression of TB. Therefore, lncRNAs have been widely expected to serve as promising molecular biomarkers and therapeutic targets for TB. In this review, we summarized the functions of lncRNAs and their regulatory roles in the development and progression of TB. More importantly, we widely discussed the potential of lncRNAs to act as TB biomarkers, which would offer new possibilities in novel diagnostic strategy exploration and benefit the control of the TB epidemic.

## 1. Introduction

Tuberculosis (TB), a chronic disease caused by *Mycobacterium tuberculosis* (Mtb), remains one of the top killers among infectious diseases. An estimated 10.6 million people worldwide were reported to have TB in 2021, a 4.5% increase from 10.1 million in 2020, reversing years of slow decline. The number of deaths officially categorized globally as due to TB (1.4 million) was more than double the number of deaths due to HIV/AIDS (650,000) in 2021, making it the single most important cause of death after COVID-19 as the second leading infectious cause of death [1]. As one of the most successful pathogens coexisting with humans, Mtb has evolved multiple immune escape mechanisms to avoid the clearance of the human immune system [2,3], which results in the harbor of Mtb in the host cells for long-term survival. The host cells, containing Mtb inside, are always imprisoned in the fiber structure wrapped granulomas [4], which therefore provides plenty of barriers to protect Mtb from anti-TB drug killings, leading to the long duration, low efficiency, strong side effects, and high susceptibility to drug resistance. Thus, how understanding the pathological and immunological mechanisms involved in TB, especially regarding a few critical biomolecules in TB development and progression, is more and more important for the effective control of TB.

In recent years, due to the outbreak of COVID-19, a large number of medical resources and attention have been used to combat the virus epidemic. As a result, the infection rate of TB increased while the diagnosis capacity was stagnant, which further increased the number of un-diagnosed TB patients as a consequence. Among various technologies for TB diagnosis in the clinic, sputum smear is one of the most classical strategies [5], which takes a couple of weeks to obtain the final results due to the low growth rate of Mtb. This time-consuming strategy in the clinic introduces multiple issues for the diagnosis of TB, especially the delay of anti-TB treatment. Additionally, the diagnosis of latent TB also remains an urgent challenge, which requires the discovery and development of new biomarkers. Thus, how to develop novel biomarkers as targets for early, rapid, and accurate detection strategy development has become one of the most important issues in TB control [6].

An increasing number of studies have shown that the expression of several specific RNAs has been associated with the occurrence, development, therapy, and prognosis of TB [7,8]. LncRNAs have been referred to as a form of RNA with more than 200 nucleotides but lack apparent open-reading frames (ORFs), meaning they cannot be translated into proteins. LncRNAs have been regarded previously as useless byproducts in the genome due to this lack of protein-encoding capacity. However, more and more research has indicated the roles of lncRNAs in covering various biological functions, like gene regulation, cell proliferation, survival, differentiation, and chromatin remodeling [9,10]. Additionally, an increasing number of studies have shown that the dysregulation of lncRNAs is associated with different diseases, such as cancer, cardiovascular diseases, autoimmune diseases, and neuroinflammation [7,8,11,12]. The deregulation of lncRNAs has also been found in different viral and bacterial infections [13,14], which highlights the potential roles of lncRNAs as biomarkers for infectious diseases.

To explore new diagnostic and therapeutic targets for more effective control of TB and drug-resistant TB in the future, lncRNAs have been widely analyzed with the development of in-depth study techniques for RNA [10,15]. A series of shreds of evidence has been presented to show the differences in lncRNAs expression and functions in innate immune responses during TB development, which indicate the potential of lncRNAs to act as a target for diagnostic, preventive, and therapeutic strategy developments [10,16]. In this review, we summarized the functions of lncRNAs and also introduced the recent progress of lncRNAs studies in TB, as well as the immunological regulation mechanisms involved in TB. More importantly, the potential of lncRNAs as TB biomarkers have also been widely discussed in this review, which may offer new possibilities in future diagnostic strategy developments.

## 2. Basic Biological Functions of LncRNAs

LncRNAs are the transcribed products of RNA polymerase II or III transcription, which can regulate gene expression under different levels in the form of RNA [17]. It is well known that the extensive transcription of higher eukaryotic genomes can produce large amounts of lncRNAs that do not encode proteins but can perform important biological functions by interacting with target DNA, RNA, and proteins [18]. Therefore, lncRNAs are able to regulate vast amounts of cellular signaling events by directly interacting with and regulating the functions of DNAs, RNAs, and proteins [19].

**Figure 1:**
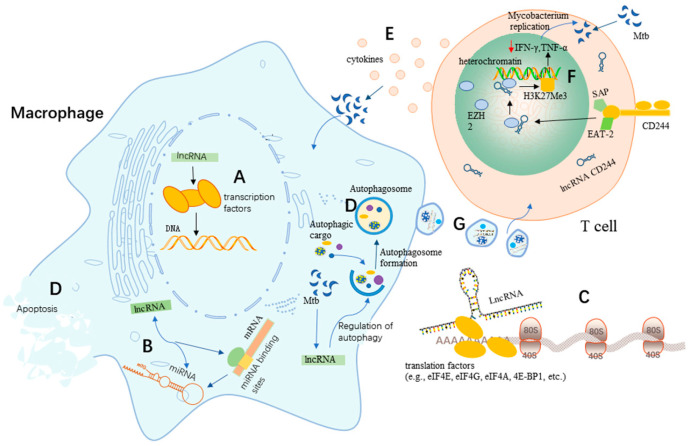
Potential roles of lncRNAs in TB. (**A**) LncRNAs regulate the transcription factors involved in DNA damage, repair, and genome stability. (**B**) LncRNAs regulate the activity of miRNAs. (**C**) Regulation of protein translation by lncRNAs. (**D**) LncRNAs regulate apoptosis and autophagy in macrophages upon Mtb infection. (**E**) T cells promote host defense by releasing Th1 cytokines to act on Mtb-infected macrophages. (**F**) A proposed model for the involvement of lncRNAs in adaptive immunity against Mtb. LncRNA-CD244 localizes in the nucleus and mediates the recruitment of the polyoma protein EZH2, which trimethylates H3K27 of the IFN-γ and TNF-α promoters, thereby inducing suppressive chromatin (heterochromatin) at the inf-g and tnf-a motifs [26]. (**G**) Exosomes mediate intercellular information exchange.

It has been found that RNAs produced from DNA regulatory elements have more general functions in facilitating transcription factor binding. The binding of transcription factors to both DNA and RNA may facilitate their association with transcriptional elements [20]. Numerous studies have shown that transcriptional behavior or DNA elements within lncRNA loci are more likely to be the source of regulatory activity than the lncRNAs themselves [21,22]. Moreover, lncRNAs are crucial players in DNA machinery, which play critical roles in DNA damage, DNA repair, and genomic stability of DNA (Figure 1A) [23,24]. DNA methylation plays a vital role in mammalian growth and development, while abnormal DNA methylation can cause disease and developmental abnormalities; studies have shown that lncRNAs play critical roles in the regulation of DNA methylation [25].

LncRNAs can not only bind to miRNAs but can also bind to the miRNA-binding sites on target mRNAs to regulate miRNA activity [27], which indicates that lncRNAs could act as regulators or effectors of miRNAs through various post-transcriptional mechanisms, thereby regulating gene expression (Figure 1B). It has been widely proven that lncRNAs can act as molecular sponges that competitively adsorb miRNAs and inhibit their regulatory effects on target genes at the post-transcriptional level [28]. For example, the lncRNA LINC00336 can suppress ferroptosis in lung cancer by functioning as a competitive endogenous RNA [29].

An emerging number of studies have revealed that lncRNAs can regulate protein translation to control signaling pathways and translation factors [19]. Numerous studies revealed that some lncRNAs have a small open reading frame (sORFs/smORFs) associated with ribosomes, suggesting the potential of lncRNAs to encode proteins [30,31,32]. A recent study showed that lncRNAs could regulate protein translation by regulating translation factors (e.g., eIF4E, eIF4G, eIF4A, 4E-BP1, etc.) (Figure 1C) [19]. In addition, it has been experimentally demonstrated that a few lncRNAs are capable of encoding small proteins (of less than 100 amino acids) called micropeptides that are involved in regulating various biological processes [33]. Based on these functions, lncRNAs play important roles in protein translation, which further regulate several important signaling pathways [19].

## 3. Roles of LncRNAs in TB

### 3.1. LncRNA-Induced Apoptosis and Autophagy Are Involved in Mtb Infection

Macrophages exhibit significant antimicrobial activity as the first line of defense against Mtb infection. A current study has shown that apoptosis and autophagy are important defense mechanisms for a variety of pathogenic bacterial infections, including Mtb [34]. Macrophage apoptosis has been widely accepted to enhance the host defense against TB via packaging the Mtb bacilli in apoptotic bodies to prevent the release of intracellular Mtb and the spread of Mtb to other healthy macrophages [34]. Meanwhile, autophagy, a critical physiological process for maintaining cellular homeostasis, can act as an innate defense mechanism in macrophages to promote intracellular Mtb killing through the formation of autolysosomes. The apoptosis and autophagy of macrophages can not only directly kill Mtb through disrupting the intracellular Mtb survival environment but can also increase the recruitment of immune cells to the TB lesion for more effective Mtb clearance. Therefore, promoting macrophage apoptosis and autophagy has been considered a novel anti-TB strategy.

Due to their critical roles in apoptosis and autophagy regulation, lncRNAs have been expected to rebuild anti-TB innate immunity by regulating apoptosis and autophagy signaling events [35]. The lncRNA MIAT has been shown to regulate autophagy and apoptosis in macrophages infected by Mtb through the miR-665/ULK1 signaling axis (Figure 1D) [35]. LncRNA MIAT expression was enhanced in BCG-infected THP1 cells, while miR-665 levels decreased in a time-dependent manner. It was found that the inhibition of MIAT effectively enhanced cell survival while limiting apoptosis. Furthermore, interference of MIAT was reported to reduce the expression of the autophagy-related markers LC3-II and Beclin-1 in THP-1 cells after BCG infection, while the expression of p62 increased to inhibit autophagy in macrophages. In addition, MIAT, as a ceRNA, negatively regulated the expression of miR-665 by affecting ULK1, a target of miR-665. Their study also indicated that the role of MIAT depletion in macrophage apoptosis and autophagy was largely attenuated by miR-665 inhibition and ULK1 enrichment. MIAT was up-regulated in Mtb-infected macrophages and could act as a negative regulator of autophagy and antimicrobial activity by manipulating the miR-665/ULK1 axis during infection, which thereby makes it a novel target for potential anti-TB drug development.

Apoptosis and autophagy can disrupt the intracellular habitat of Mtb, which in turn triggers innate and adaptive immune responses [36,37]. A recent study showed that long intergenic non-coding RNA-erythroid pro-survival (lincRNA-EPS) played an important role in the regulation of apoptosis and autophagy in macrophages (Figure 1D) [38]. It indicated that the expression of lincRNA-EPS was significantly down-regulated in monocytes from patients with active pulmonary tuberculosis (PTB) compared to healthy individuals. It also found that the low expression of lincRNA-EPS could regulate apoptosis and autophagy through the JNK/MAPK signaling pathway. In this study, the knockdown of lincRNA-EPS was found to inhibit apoptosis and enhance autophagy in BCG-infected RAW264.7 macrophages. Thus, these findings suggest that lincRNA-EPS has a greater potential to become a new diagnostic marker for PTB as well as a therapeutic target.

Emerging studies have suggested a potential regulatory role for lncRNAs in the host immune response to Mtb infection [39]. Aberrantly expressed lncRNAs have been observed in human macrophages infected with Mtb [40,41]. For example, the lncRNA PCED1B-AS1, an endogenous sponge, was found to be involved in apoptosis and autophagy in Mtb-infected macrophages (Figure 1D) [39]. PCED1B-AS1 expression was significantly reduced in CD14+ monocytes from patients with active TB (*p* < 0.05). The knockdown of PCED1B-AS1 in monocyte-derived macrophages (MDMs) and THP-1 derived macrophages (TDMs) inhibited TNF-α-induced apoptosis with enhanced macrophage autophagy, suggesting that PCED1B-AS1 could act as a regulator of macrophage apoptosis and autophagy. Meanwhile, these researchers also found that PCED1B-AS1 could serve as an endogenous sponge to block miR-155 expression in macrophages by directly binding to miR-155. Moreover, the overexpression of the target gene for miR-155, FOXO3/Rheb, could reverse the effects of PCED1B-AS1 on apoptosis and autophagy in macrophages [39]. Therefore, PCED1B-AS1 is involved in macrophage apoptosis and autophagy in active TB by targeting and regulating the miR-155/FOXO3 (Rheb) axis, highlighting PCED1B-AS1 as a new diagnostic marker for TB as well as a target for the development of potential therapeutic interventions against TB.

A current study noted that lincRNA-Cox2 expression was up-regulated in BCG-infected RAW264.7 cells, and that the apoptosis of BCG-infected RAW264.7 cells was promoted through the knockdown of lincRNA-Cox2 [42]. LincRNA-Cox2 was found to be a mediator of the activation and inhibition of immune gene expression in innate immune cells [43]. It was further found that lincRNA-Cox2 enhanced the ability of M1 macrophages to promote apoptosis and decreased the ability of M2 macrophages to inhibit apoptosis [44]. Further investigation of the regulatory role of lincRNA-Cox2 in macrophage apoptosis may provide new ideas for the development of new diagnostic or therapeutic strategies for TB.

### 3.2. Association of LncRNA Gene Polymorphisms with the Susceptibility to TB

Earlier research studies have validated that multiple lncRNA genetic mutations were linked to the genetic susceptibility towards PTB [45,46]. A recent study found that the level of NEAT1 expression was anomalously high in the peripheral blood mononuclear cells of patients diagnosed with PTB [47]. This study examined the correlation between single nucleotide polymorphisms (SNPs) located within the NEAT1 gene and PTB susceptibility. These researchers used improved multiple ligase detection reactions (iMLDR) to conduct genotype analysis for four SNPs (rs2239895, rs3741384, rs3825071, and rs512715) of the NEAT1 gene in a cohort consisting of 479 individuals diagnosed with PTB and 476 controls. Their results showed that the genotype and allele frequencies of the four SNPs did not differ significantly between the PTB patients and controls (all *p* > 0.05), nor did they differ statistically significantly from the genetic susceptibility of PTB patients (all *p* > 0.05). In comparison to the sputum smear-negative PTB patients, the frequencies of the TT genotype and T allele in the rs3825071 polymorphism significantly rose among the sputum smear-positive PTB patients (*p* = 0.010 and *p* = 0.003, respectively). Consequently, there could be an association between the rs3825071 polymorphism and sputum smear-positive result amongst PTB patients.

The importance of single nucleotide polymorphisms (SNPs) located in lncRNAs as critical regulators has now been widely recognized [48]. A case study in a western Chinese population showed that the expression levels of lnc-TGS1-1 and its variant rs4737420, as well as lnc-AC145676.2.1-6 and its variant rs111352767 were associated with the susceptibility to TB and therefore had important clinical implications for TB diagnosis and therapy [49]. Lnc-AC145676.2.1-6 and lnc-TGS1-1 were both significantly down-regulated in TB patients, suggesting that lnc-AC145676.2.1-6 and lnc-TGS1-1 may somehow reduce the risk of Mtb. In addition, the low expression of lnc-TGS1-1 during anti-TB treatment was associated with thrombocytopenia in TB patients, and the homozygous CC genotype of rs4737420 was associated with leukocytes in the dominant mode compared to individuals with the T allele (TT/CT genotype) [49]. Li et al. identified the key role of the long intergenic non-coding RNA LINC00152 in TB, which indicated that LINC00152 expression was down-regulated in TB patients compared to healthy individuals (*p* < 0.001) [50]. Furthermore, it was found that LINC00152 expression levels were at their lowest in rs80292941 TT genotype carriers among TB patients (p = 0.04), whereas rs80292941 AA genotype carriers had a higher susceptibility to anti-TB drug-induced hepatotoxicity (*p* = 0.002) [50]. These findings suggest that lncRNAs are closely associated with the development of TB, and that lnc-TGS1-1 might be able to serve as a potential predictor or novel susceptibility marker for anti-TB drug-induced hepatotoxicity.

LncRNA gene polymorphisms have also demonstrated their potential to distinguish between active TB and latent TB. A current experimental study found that the lncRNA transcript AC079767.4 (Ensembl ID ENST00000429730), encoded by gene AC079767.4 (Ensembl ID ENSG00000224137), was down-regulated in both the latent TB and active TB groups compared to the healthy controls [51]. Additionally, AC079767.4 was expressed at elevated levels in latent TB patients compared to active TB patients [52], which indicated that the lncRNA AC079767.4 gene was likely to be involved in the development and progression of TB infection. It was also found that the single nucleotide polymorphisms (SNPs) of the lncRNA AC079767.4 showed an essential impact on the clinical phenotype and susceptibility to TB disease in the Han Chinese population in western China [51]. Patients with the CC genotype of rs12477677 among four SNPs of the AC079767.4 gene were associated with a reduced incidence of fever (*p* = 0.016); the C allele was associated with a reduced susceptibility to TB (*p* = 0.026, but Bonferroni corrected *p* = 0.103), while the T allele was associated with lower levels of the erythrocyte sedimentation rate (ESR) in the dominant model of rs1055229 (*p* = 0.021) [51]. Together, these findings suggested that the C allele of rs12477677 within the lncRNA AC079767.4 gene might have relatively weak protective effects against the development of TB. Furthermore, haplotype analysis of these genetic variants revealed that the ACAC haplotype was formed by the four AC079767.4 polymorphisms, and that the potentially beneficial C allele in the rs12477677 locus slightly reduced the risk of TB (*p* = 0.045), which was consistent with the SNP analysis results [51]. However, we found that except for the results of fever, all the obtained *p*-values for these results were relatively high with extremely small differences. Thus, although these data suggested the protective potential of the C allele in rs12477677 on the development of TB, more detailed work is needed to further confirm these effects and explore the precise mechanisms.

Studies have shown that CD8+ T cells could promote the host defense by either releasing Th1 cytokines or through directly killing the Mtb-infected macrophages (Figure 1E) [53]. Emerging studies have found that among the differentially expressed lncRNA profiles, lncRNA transcripts encoded by the gene CASC8 (Ensembl ID ENSG00000246228) are highly expressed in the CD8+ T cells under active TB compared to healthy controls, suggesting that the lncRNA CASC8 may be involved in the development of TB infection [53]. Subtype analysis showed that the SNP rs7836840 C allele of the lncRNA CASC8 was significantly associated with TB susceptibility (OR = 1.196, 95% CI = 1.05–1.362, *p* = 0.02739 after Bonferroni correction). Thus, genetic polymorphisms of lncRNAs might also be important for TB susceptibility, which further enhances the potential of lncRNAs to be developed as potential biomarkers for the development of TB. Although increasing pieces of evidence have indicated that there was an association between some lncRNAs and MTB susceptibility, there were still no formal results to demonstrate that lncRNAs directly regulate MTB susceptibility. Thus, more work is needed to further explore the precise mechanism involved in the association between lncRNAs and MTB susceptibility, which might provide novel targets for anti-TB strategy development.

### 3.3. LncRNAs Regulate the Immune Response during Mtb Infection

Bacterial interactions with host cells lead to alterations in the host transcriptional program associated with epigenetic mechanisms [54]. It has been shown that the transcriptome of innate immune cells is altered after Mtb infection, depending on the regulation of non-coding RNAs, DNA methylation, or histone modifications [55]. The reprogramming of the transcriptional program may lead to more effective immune responses or allow the pathogen to exploit the host’s mechanisms for its own benefit, where lncRNAs may play critical roles. It was shown that the lncRNA HOTAIR could regulate the expression of DUSP4 and SATB1 during Mtb infection [55]; additionally, it was found to be able to bind to and target the H3K27 methylesterase complex PRC2 in different sites, thereby altering the trimethylation of H3K27 [56]. Thus, the epigenetic modification of host cells plays a vital role in the innate immune response against TB [57,58].

In recent years, there has been increasing interest in the roles of lncRNAs in macrophage regulations upon Mtb infection [59]. Subuddhi et al. found that the lncRNA HOTAIR was down-regulated in H37Rv-infected THP-1 macrophages, which increased the transcription of dual-specificity phosphatase 4 (DUSP4) and SATB homeobox 1 (SATB1) to promote the survival of Mtb in macrophages [55]. Moreover, it was worth noting that the lncRNA HOTAIR was upregulated in H37Ra-infected macrophages, but downregulated in H37Rv-infected macrophages, which suggested that the reduction in the lncRNA HOTAIR in infected macrophages was specific for the virulent strain of Mtb. Importantly, these results highlighted the potential use of the lncRNA HOTAIR as a Mtb-specific biomarker that might not be disturbed by other kinds of bacterial infections. However, more work is needed to further explore these potential uses.

Immune responses were also associated with the expression of different lncRNAs due to their roles as trans-acting regulators of protein-coding genes [60,61]. Through TLR1/2 stimulation of THP1-derived macrophages, the lncRNA THRIL was found to form a complex with the ribonucleoprotein (RNP) hnRNPL, which acted on the TNF-α promoter to trans-regulate TNF-α expression [62]. In another report, the lncRNAs BAIAP2, MARCKS, and BCAT1 were all shown to regulate the expression of inflammatory genes in macrophages stimulated by Toll-like receptor (TLR) ligands [63]. These findings collectively suggested that the expression of lncRNAs in macrophages strongly contributed to the host cell immunity against TB infection.

### 3.4. Inflammatory Regulations of LncRNAs against Mtb Infection

The inflammatory response is an essential issue of TB as Mtb can evade the host’s immune response, which in turn causes inflammation in the lungs [64,65]; therefore, suppression of the inflammatory response is also critical to treating TB. Several lncRNAs have been shown to be involved in TB-associated inflammation and therefore influence the progression of TB [66]. Studies have shown that the lncRNA CCAT1 can promote the inflammatory response by regulating the miR-185-3p/MLCK signaling pathway, which is strongly activated to destroy barrier function and promote the pathogenesis of inflammatory bowel disease [67]. Due to the crucial roles of inflammatory responses in TB development, the lncRNA CCAT1 might therefore be also involved in TB. The latest study found that the lncRNA CCAT1 was up-regulated in patients with both recurrent tuberculosis (R-TB) and newly developed tuberculosis (N-TB) compared to healthy individuals (*p* < 0.05). Plasma CCAT1 levels decreased consistently with the treatment of TB (*p* < 0.05), which indicated that CCAT1 expression might reflect the outcome of TB treatment. In addition, this study noted that high plasma CCAT1 levels reported on the day of admission were strongly associated with a poorer survival in patients with R-TB and newly developed TB [68]. Therefore, testing for CCAT1 on the day of admission may help to identify patients at high risk of death and thus assist clinicians in developing treatment strategies to improve patient survival.

Recent studies have revealed that lincRNAs, a long non-coding transcript from the intergenic region of annotated protein-coding genes, are involved in regulating the inflammatory response of Mtb-infected macrophages [69]. A noteworthy example is the lincRNA cyclooxygenase 2 (Cox2), an early primary response gene that has a regulatory role in the expression level of NF-κB in macrophages [69]. LincRNA-Cox2 expression was up-regulated in both the plasma and monocytes of TB patients compared to healthy controls. Studies have shown that NF-κB consists of homologous or heterologous complexes of NF-κB subunits, including human p65, c-Rel, RelB, p52, and p50, and is an important regulator of inflammation [70]. Knockdown of lincRNA-Cox2 in H37Ra-infected macrophages revealed a significant reduction in the inflammatory regulatory proteins NF-κB and Stat3, suggesting that lincRNA-Cox2 may be required to activate NF-κB and Stat3 to regulate the inflammatory response involved in resistance to TB [69]. Thus, lincRNA-Cox2, as a key mediator in the development of inflammatory responses, was expected to be a new biomarker for diagnosing and treating TB.

The down-regulation of GAS5 could modulate the inflammatory responses of Mtb-infected THP-1 cells, which was deemed as valuable for the diagnosis of TB [71]. The expression of the lncRNA GAS5 was down-regulated in the sera of TB patients compared to healthy controls and was at its lowest level in patients with active TB (*p* < 0.001). Bioinformatic analysis revealed that GAS5 was negatively correlated with the concentration of serum inflammatory factors, suggesting that the expression level of GAS5 had important influences on the degree of TB inflammation. It was confirmed that the overexpression of GAS5 enhanced the conversion of the anti-inflammatory M2-subtype macrophages into the pro-inflammatory M1 subtype [72]. It was further found that the overexpression of GAS5 reversed the enhanced macrophage viability and inflammatory activation induced by Mtb infection, which indicated that regulating GAS5 expression may help in controlling the development of TB.

Mtb can travel with the blood and lymphatic circulation to different parts of the body to form extra PTB [73]. For example, bone TB, a destructive lesion caused by Mtb invading either the bone or joint [74], usually occurs in the spine, knee, foot, and hand, which severely impacts the weight and movement of the joint. The spine is the most prevalent site for extra PTB, accounting for more than half of the incidence of osteoarticular TB along with a high rate of disability, which causes significant pain and reduces the quality of life of patients [75,76]. The expression level of the lncRNA SNHG15 was found to be significantly increased in spinal TB tissues compared to healthy controls (*p* < 0.05), where SNHG15 suppressed the secretion of inflammatory cytokines by regulating the RANK/RANKL pathway to affect osteoclasts [77]. Osteoclasts can not only partially affect the resistance to Mtb, but also allow Mtb to survive in vivo, thus promoting latency and infection [78,79]. It was further found that Mtb could reduce osteoclast apoptosis and attenuate the osteoblast responses to stimulation, thus promoting the escape of Mtb and counteracting the bactericidal effect of immune cells [80]. The RANKL and RANK protein has been shown to be the most important pair of ligands and receptors in the osteoblast signaling system and increasing their expression can promote osteoblast formation and proliferation [81,82]. The down-regulation of tuberculin through transfection with SNHG15 siRNA inhibited osteoclast proliferation and resulted in a decrease in the RANK and RANKL genes and protein [77], indicating the roles of lncRNAs in regulating the secretion of inflammatory factors in extra PTB.

Interestingly, the roles of lncRNAs in inflammatory responses can also be regulated using drugs in Mtb infection. Numerous studies have found that lncRNAs could drive their functions through cis-regulating proximal genes and trans-regulating distal genes located on other chromosomes, which were key factors in regulating immune cell development and function [83,84]. It was shown that Mtb infection induced p50-associated COX-2 extragenic RNA (PACER) and lincRNA-p21 expression, while treatment with triptolide before or after Mtb infection led to the Mtb-induced expression of these lncRNAs in monocyte-derived macrophages [66]. Investigations revealed that triptolide, as a diterpenoid tricyclic oxide, was one of the active compounds of the herb Tripterygium wilfordii Hook f., which exerted biological activities, such as anti-inflammatory, anti-tumor, and immunoregulation by inducing apoptosis, remodeling of the epigenetic structure of target cells, and targeting pro-inflammatory cytokines [85,86]. Furthermore, it was found that the expression of these lncRNAs was inversely related to their target genes and that they mediated, at least partially, the cytotoxic and anti-inflammatory activities of triptolide in resting and activated monocyte-derived macrophages [66]. Triptolide was reported to regulate Ptgs-2 expression in quiescent monocyte-derived macrophages mainly through a lncRNA PACER-independent mechanism, and that the lncRNA PACER could act as a negative regulator of Ptgs-2 in quiescent monocyte-derived macrophages similar to that of lincRNA-Cox2 [66]. Further studies revealed that Ptgs-2 and IL-6, the target genes of the lncRNA PACER and lincRNA-p21, were pro-inflammatory genes that played a vital role in the cellular immune response to kill Mtb [87,88]. Previous reports have indicated that Mtb hijacks host non-coding RNAs to evade the immune response, which helps the Mtb to persist within the infected host cells [26,89]. However, PACER was found to be able to promote macrophage activation toward a pro-inflammatory M1 phenotype, which effectively killed Mtb as a result [44]. Thus, in resting and Mtb-infected human macrophages, triptolide regulates the expression of the lncRNA PACER and lincRNA-p21, as well as their target genes IL-6 and Ptgs-2 and could also inhibit the growth of Mtb in monocyte-derived macrophages. In conclusion, these results suggest that lncRNAs play essential roles in the inflammatory responses during TB infection, which, therefore, can be developed as inflammation-associated biomarkers or therapeutic targets in TB.

## 4. LncRNAs as Potential Biomarkers of TB

In recent years, the progressive increase in the incidence of extensively drug-resistant TB has brought new challenges to TB prevention and treatment, making TB treatment more difficult and a serious risk to human health [90]. The early, rapid, and accurate diagnosis of TB has thus become one of the most emergent issues for better control of TB epidemics. Due to the critical roles and advanced functions of lncRNAs in the TB immunity, lncRNAs, therefore, show potent potential as novel biomarkers for TB, which is expected to be explored for potential TB diagnosis applications based on different sample sources.

RNA is less stable and easily degraded for three main reasons, including the following: (i) RNA is single-stranded, which makes it easier to be attacked by enzymes [91]; (ii) RNA has a highly reactive hydroxyl group on C2 that takes part in RNA-mediated enzymatic events; and (iii) enzymes that degrade RNA (ribonucleases) are abundant in the environment and are difficult to remove completely. For example, autoclaving a solution containing bacteria destroys the bacterial cells but not the ribonucleases released from the cells. In addition, even trace amounts of ribonucleases can degrade RNA. Extracellular RNA was first identified in plasma and serum over 70 years ago [92]. Due to the presence of ribonucleases in the extracellular space as well as in body fluids, it was widely believed for many years that RNA could not exist stably outside the cell and that extracellular RNA was rapidly degraded upon its release. It was not until the publication of two groundbreaking studies by the scientists Ratajczak and Valadi et al. that demonstrated the presence of RNA in microvesicles and exosomes, and that these RNAs could be secreted outside the cell and act as signaling molecules to influence the behavior of the recipient cell. This process can occur both between neighboring cells and can be regulated over long distances [93,94]. Subsequently, extracellular RNA was found in almost all body fluids, such as blood, urine, sputum, cerebrospinal fluid, pleural fluid, etc. [92]. In addition, high-throughput sequencing has also shown that in addition to mRNA, extracellular RNA contains a variety of non-coding RNA types, such as miRNA, piRNA, tRNA, lncRNA, and so on [95]. Therefore, lncRNA can be identified in specimens, such as in the serum and sputum, rather than being detected due to the degradation of lncRNA into small fragments.

In the following portions, we analyzed the lncRNAs that could be potential biomarkers for diagnosing tuberculosis, and basic information on these lncRNAs has been summarized in Table 1. The advantages and challenges of lncRNA biomarkers compared to current clinical tests for TB have summarized in Table 2.

### 4.1. LncRNAs from Peripheral Blood as a Potential Biomarker for TB

Blood samples for clinical testing are straightforward to be obtained compared with tissue samples and can be monitored dynamically with little pain for patients. The direct use of peripheral blood samples for testing is more convenient and faster than using PBMCs or plasma samples, which do not require further isolation and purification.

It is well known that after Mtb infection, the host’s immune system initiates a series of immune responses to fight against Mtb infection. The host immune response provides a new arsenal to screen for potential markers that can be applied for the diagnostic studies of TB by analyzing the peripheral blood samples. For example, AC007128.1, a lncRNA transcribed from chromosome region 7p21.3, was found to be significantly associated with susceptibility to active TB in the chromosomal region 7p22-7p21 (*p* = 0.0002) by association analysis of a whole microsatellite genome scan, which suggested that AC007128.1 might be strongly associated with the development of TB [96,112]. This study suggested that AC007128.1 might contribute to the up-regulation of mRNA, which blocks the G protein-coupled receptor signaling pathway that elicits the host immune response and inflammation in TB patients [96]. Furthermore, comparing the lncRNA expression profiles of active TB patients vs. latent TB patients vs. healthy controls using peripheral blood samples, the expression levels of AP001065.3 (Kruskal–Wallis; *p* = 0.018) and AC007128.1 (Kruskal–Wallis; *p* = 0.012) were found to be significantly different. It indicated that AP001065.3 and AC007128.1 could be used as biomarkers to distinguish active TB infection from latent TB infection. Meanwhile, the expression of AC700128.1 was found to be specific and significantly up-regulated in TB patients through external data authentication, suggesting that AC700128.1 has a vital role in the pathogenesis of TB and has the potential to be a specific biomarker for the diagnosis of TB [96].

The use of lncRNAs in peripheral blood samples as potential biomarkers provide the possibility for the development of a novel TB diagnostic strategy. However, peripheral blood has a short shelf life, and if it is not used in time, it needs to be combined with preservatives to prevent clotting and keep the cellular components fresh, along with refrigeration to avoid bacterial infections. In addition, as Mtb infections always arise in the lung, the concentration of the target biomarker, such as lncRNAs in the blood, is usually low, which may therefore require a large number of samples and the development of highly sensitive detection methods.

### 4.2. LncRNAs from Purified PBMCs as Potential Biomarkers in TB

PBMCs refers to peripheral blood mononuclear cells, which mainly include lymphocytes, monocytes, phagocytes, dendritic cells, and a small number of other cell types. In whole blood, erythrocytes and polymorphonuclear cells are the main cells that show a much higher proportion than mononuclear cells. These cells’ presence can significantly impact the diagnostic results, which might introduce incorrect results. In contrast, purified PBMCs can easily mimic the blood immune environment in vitro, which has since become a necessary measure for the clinical diagnosis of different diseases. Only a few separation operations are required to purify PBMCs from the whole blood, which clinical laboratories can easily satisfy.

Taking advantage of the sensitivity of PBMC samples, lncRNAs can also be analyzed as biomarkers for potential TB diagnosis. The lncRNA NEAT1 (including NEAT1_1 and NEAT1_2) has been shown to be expressed at relatively higher levels in TB patients than that in controls, while there was no significant difference observed in their expression between the new case group and the relapse TB case group [97]. The expression of NEAT1 was significantly increased in PBMCs from TB patients and its expression level gradually decreased with anti-TB therapy until it returned to normal levels. Furthermore, during Mtb infection, NEAT1 affected cytokine expression in macrophages via the JNK/ERK MAPK pathway [97] and was involved in the up-regulation of TNF-α and IL-6 in the inflammatory responses, which largely influenced the outcome of TB as a result. In addition, the knockdown of NEAT1 led to a significant increase in the quantity of bacterial CFUs in infected THP1 cells, suggesting that NEAT1 was required for the killing of Mtb [113]. As the dynamic changes of NEAT1, to some extent, reflect the anti-TB treatment effects, further understandings of the molecular mechanisms and biological functions of NEAT1 in TB are expected to provide new targets for the diagnosis and treatment of TB.

It is widely known that the interaction between macrophages and Mtb alters the gene expression profile of cells. Mtb evades the bactericidal effects of macrophages through various bacterial immune subversion mechanisms and survives using various intracellular resources [3,114]. It has been found that different strains of Mtb can induce different host responses leading to different outcomes [115]. For example, infection with virulent Mtb results in macrophage necrosis and pathogen spread to surrounding tissues, whereas with infection with nonvirulent Mtb, macrophages are predisposed to apoptosis for Mtb clearance [116]. Researchers have identified four differentially expressed lncRNAs using microarray analysis [98]. qPCR was performed to examine the expression levels of these four lncRNAs in PBMC samples from PTB patients and healthy individuals vaccinated with BCG, including MIR3945HG V1, MIR3945HG V2, ENST00000360485, and ENST00000417932. qPCR results showed that compared with healthy controls, the expression levels of MIR3945HG V1 and MIR3945HG V2 were significantly higher in PTB patients (*p* < 0.001), and the expression of ENST00000360485 was down-regulated (*p* < 0.001), while the expression of ENST00000417932 was not significantly changed. In addition, the AUC (area under the ROC curve) of ENST00000360485 was found to be lower than 0.8 by ROC data analysis, while the AUCs of MIR3945HG V1 and MIR3945HG V2 exceeded 0.9, respectively, suggesting that MIR3945HG V1 and MIR3945HG V2 had more substantial potential to become novel biomarkers for the diagnosis of TB.

Misdiagnosis of pulmonary tuberculosis (PTB) in the absence of pathogenic, diagnostic evidence is still a major issue for this disease. Currently, more and more researchers are constructing a potential predictive model that targets lncRNAs to diagnose PTB. A recent study analyzed the expression levels of the lncRNA n344917 using PBMCs from PTB patients, and then combined electronic health record (EHR) information to construct a predictive model [99]. The results of this study showed that this model had good clinical usability, consistency, and discriminative power (specificity = 86.43%, sensitivity = 88.98%, cutoff = 0.657, negative predictive value = 89.63%, positive predictive value = 85.61%, and area under the curve (AUC) = 0.88). In addition, microarray analysis revealed that n344917 expression was significantly reduced in the PBMCs of patients with clinically diagnosed PTB (*p* < 0.05). Meanwhile, NONCODE database analysis showed that n344917 was up-regulated in lymphocytes as well as leukocytes, indicating its potential involvement in the immune response against Mtb infection. Therefore, n344917 may serve as a novel molecular biomarker for the clinical diagnosis of PTB, which is beneficial for anti-TB strategy development.

Recent studies have investigated the potential of lncRNAs and their associated predictive models for diagnosing PTB patients. One study found that clinically confirmed TB patients had differing ENST00000497872, n333737, and n335265 expression levels [100]. These researchers devised a nomogram to accurately predict the infection that utilized data on all three lncRNAs and six electronic health records (EHRs), including age, hemoglobin, weight loss, low-grade fever, calcified foci on imaging, and interferon-gamma release test. The nomogram exhibited an AUC of 0.89, a sensitivity of 0.86, and a specificity of 0.82, displaying a better discriminatory ability than the EHR models in distinguishing PTB patients from non-TB disease controls. Therefore, the nomogram of the “LncRNA + HER” model is anticipated to be efficacious in the timely diagnosis of tuberculosis under clinical settings. Similarly, Chen and his team screened two novel specific lncRNA markers (TCONS_00001838 and n406498) for TB in host PBMCs using Affymetrix HTA2.0 array and qRT-PCR, with statistical significance (*p* < 0.001) [101]. They combined these two differentially expressed lncRNAs with eight EHR parameters through logistic regression models and nomogram visualization, obtaining the best model, “LncRNA+EHR”, with an accuracy of 0.79 (0.75–0.82), a sensitivity of 0.81 (0.78–0.86), a specificity of 0.73 (0.64–0.79), and an AUC of 0.86. Consequently, the lncRNAs TCONS_00001838 and n406498 also have the potential to become new molecular markers for the diagnosis of PTB.

Numerous studies have shown that CD4+ T cells, CD8+ T cells, and γδ T cells are essential for the immune responses against Mtb infection [117,118,119]. Mtb infection stimulates the expression of lncRNAs in CD4+ T cells to varying degrees, thereby modulating the host immune response to Mtb infection. Also, Mtb infection could induce the differential expression of T cell suppressor molecules on CD8+ T cells, which may induce impairment of CD8+ T cell immunological function [26]; the researchers of this study used PBMCs as their assay sample and found that the expression of CD244 and CD244 signaling-related molecules was up-regulated in CD8+ T cells from patients with active TB disease. Furthermore, CD244 expression/signaling in TB was associated with high levels of CD244 expression in the CD244+CD8+ T cell subpopulation. It was shown that CD244 could physically interact with the chromatin-modifying enzyme Zeste homolog 2 (EZH2), mediate H3K27 trimethylation at the IFN-γ/TNF-α locus toward a repressive chromatin state and suppress IFN-γ/TNF-α expression in CD8+ T cells (Figure 1F). These experimenters found that the expression of IFN-γ and TNF-α was significantly enhanced by knocking down the lncRNA CD244, while the protective immunity of CD8+ T cells against TB was also significantly enhanced. These results indicate that the suppression of IFN-γ/TNF-α in CD8+ T cells could be reversed by knocking down CD244. Furthermore, CD244 signaling can regulate the effector functions of CD244+CD8+ T cells during active Mtb infection using lncRNAs and histone-modifying enzymes [26]. In conclusion, CD244 and the lncRNA CD244 axis present an epigenetic program of lncRNA-driven T cell immunity against Mtb infection in the regulation of IFN-γ and TNF-α expression. Thus, CD244 may be a potential target for the treatment of TB with essential implications for the development of new vaccines and therapeutic approaches as well as for clinical diagnoses.

### 4.3. LncRNAs from Plasma as Potential Biomarkers in TB

In the current clinical test of blood samples, most parameters are analyzed in plasma except for a few specific indicators. Plasma is a cell-free supernatant that can be obtained through the centrifugation of blood with an anticoagulant. The physiological components in plasma are closer to the intertissued fluid of the body, which can reflect the physiological situation of the body with a higher reality and sensitivity. Moreover, the analysis of plasma samples can also avoid the potential errors introduced by the complicated cell components in whole blood. Therefore, plasma samples can be a better choice for screening differentially expressed lncRNAs in TB patients.

A standard gold test to define TB cure is still lacking so far, which may lead to the early discharge of TB patients, thus increasing the risk of TB transmission, recurrence, and drug resistance. Studies on biomarkers for curing TB are still at the laboratory research stage and have not been applied to clinical practice [120], which urges researchers to explore more potent biomarkers that may contribute to the potential diagnosis. Up to now, a few lncRNAs from plasma have demonstrated the potential to be effective and accurate in assessing TB cure. In a recent study, differentially expressed lncRNAs in the plasma of untreated and cured TB patients were screened using the mRNA–lncRNA–miRNA interaction network and coding-noncoding gene co-expression network analysis to predict the target genes of lncRNAs [102]. They found that the expression of the lncRNAs uc.48+ and NR_105053 was significantly different between the untreated and cured TB groups. This result suggested that the plasma lncRNAs uc.48+ and NR_105053 could be used as potential novel biomarkers of cured TB to differentiate TB patients from cured TB patients, and also indicated that plasma lncRNAs might serve as more straightforward and more effective biomarkers for potential TB analysis.

A recent study found that SOCS3, a negative regulator, played an important role in the cytokine response induced by Mtb infection, and its neighboring lncRNA XLOC_012582 was highly expressed in the B cells of patients with active TB, providing new insights into the pathogenesis of TB [121]. In addition, this study also examined the expression levels of LOC152742 in active TB, obsolete TB, BCG patients, and normal human plasma, respectively, using real-time PCR [103]. The sensitivity, specificity, and AUC of LOC152742 were 93.61%, 86.21%, and 0.923 for the plasma of active TB patients, 84.32%, 83.51%, and 0.852 for obsolete TB patients, and 80.05%, 75.92% and 0.874 for BCG-infected individuals, respectively [103]. These results indicated that LOC152742 demonstrated a high sensitivity and specificity in the plasma of patients with active PTB and could serve as a potential marker for the diagnosis of active PTB.

The detection of specific biomarkers in the plasma of TB patients at the molecular level provides new insights for the rapid diagnosis of TB [15]. Currently, molecular biomarker-based TB diagnosis has been constructed, and this assay may become the key to the rapid diagnosis of TB with high specificity and sensitivity [122]. Recent studies have revealed that many lncRNAs have a conserved secondary structure in subcellular localization and can form structural features, such as a spliced poly A tail, which may make the different expressions of lncRNAs biologically meaningful [123,124]. A notable example is ENST00000354432, an antisense transcript targeting sodium/glucose cotransporter 5 heterodimers 1 (SLC5A10), which was found to be up-regulated in the plasma of patients with active PTB (*p* < 0.05) [15]. ENST00000354432 was reported to block the transcription of SLC5A10 and its dysregulated expression was associated with renal dysfunction, which always occurs in TB infection [15,125]. Similarly, ENST00000427151, an antisense transcript targeting the CD81 antigen, also known as CD81 antisense RNA 1 (CD81-AS1), was also up-regulated in the plasma of patients with active PTB (*p* < 0.05) [15]. Additionally, an increase in the amount of the lncRNA LINC00870 present in both the plasma and sputum samples of individuals diagnosed with TB and latent TB was also observed. However, following a three-month course of anti-TB therapy (ATT), the level of LINC00870 declined, indicating its potential as a biomarker for the assessment of TB diagnosis and treatment efficacy [104].

A current study analyzed lncRNAs in plasma and their potential diagnostic value for TB using microarray analysis and RT-qPCR, which identified four differentially expressed lncRNAs [105]. The results of this study showed that compared to healthy controls, the expression levels of NR_003142 (*p* < 0.05), NR_038221 (*p* < 0.01), and ENST00000570366 (*p* < 0.05) were all significantly enhanced in the plasma of TB patients, while the expression level of ENST00000422183 (*p* < 0.001) was attenuated [105]. Meanwhile, ROC curve analysis showed that the measured AUCs of these four lncRNAs were NR_038221 (0.677), NR_003142 (0.657), ENST00000570366 (0.672), and ENST00000422183 (0.738), respectively. In addition, evaluating these four lncRNAs as models for diagnosing TB using logistic regression revealed an AUC value of 0.845, sensitivity = 79.2%, and specificity = 75%. Thus, the above experimental results suggested that the combination of these four lncRNAs was superior to a single marker in the assessment of TB. Further studies revealed that NR_038221 was most significantly associated with TB and may be involved in pathological processes during TB infection by regulating intracellular signaling pathways and signal transduction between immune cells. In conclusion, these results strongly suggest that lncRNAs could serve as potential biomarkers for the diagnosis of TB and provide a new basis for the early diagnosis of TB.

### 4.4. LncRNAs from Tissue as Potential Biomarkers in TB

In the diseased tissue samples, the target biomarkers to be tested are usually denser than that in other samples, such as in the blood or sputum, giving a higher rate of positive results and more accurate clinical diagnoses. In addition, the use of tissue samples also provides a more accurate analysis of gene expression in TB patients, as TB lesions could directly reflect the status of disease development.

It was shown that the lncRNAs ENST00000429730.1 and MSTRG.93125.4 were associated with metabolic activity in tuberculous lesions from sputum-negative PTB patients [106]. This work stated that the expression levels of both ENST00000429730.1 and MSTRG.93125.4 were up-regulated in lung tissue samples, and further mechanism studies indicated that CCL5, CXCL9, CYBB, and HLA-DMB were the predicated trans-target genes of ENST00000429730.1 and MSTRG.93125.4 [106]. Currently, CCL19 and CCL5 have been shown to be involved in chemokine signaling pathways and cytokine–cytokine receptor interactions. In addition, chemokines and cytokines were found to play a critical role in cell initiation, sequential recruitment, and activation in Mtb-infected lungs [126]. Differentially expressed lncRNAs may influence the chemokine signaling pathways and cytokine–cytokine receptor interactions through regulating their target genes in lung tissue samples [106]. Thus, ENST00000429730.1 and MSTRG.93125.4 may affect the chemokine signaling pathways and cytokine–cytokine receptor interactions by regulating their trans-target genes. ENST00000429730.1 and MSTRG.93125.4 are thereby expected to be potential biomarkers of metabolic activity of TB foci in sputum-negative TB patients.

However, tissue sample acquisition is an immensely invasive process that can always cause injuries, which is always challenging to be accepted by patients. Additionally, tissue sample acquisition is unsuitable for dynamic monitoring, which cannot provide real-time information in short periods. Sampling bias is unavoidable when obtaining tissues through punctures, regardless of which part of the patient is left. Therefore, although the tissue sample may provide more accurate information on the TB status through lncRNA analysis, more considerations should be gained in the final decision about whether to analyze these tissue samples or not.

### 4.5. LncRNAs from Sputum as Potential Biomarkers in TB

Currently, the gold standard diagnostic strategy for TB is the sputum smear, an effective way to check for the existence of Mtb. However, the low growth rate of Mtb always needs several weeks to obtain a final result, which would dramatically delay the anti-TB treatment for positive patients as a result. Although sputum smears are effectively used in the diagnosis of TB in clinical medicine, in practical terms, sputum smears are also susceptible to various factors that can lead to missed tests in the search for Mtb, such as the quality of sputum sample retention. The collection of intense cough sputum samples can lead to a high positive detection rate and, of course, high accuracy. However, deep cough sputum samples are difficult to collect due to the tested patients’ compliance, which would lead to false-negative results with a sputum smear test.

Sputum samples are mucus that is coughed up from the lower airways of patients, which can also be applied for disease diagnosis in several other respiratory diseases. The sputum of potential TB patients has always been used for sputum smear analysis, which can not only provide the bacterial shreds of evidence of the samples as the standard golden test but can also be applied for long intergenic non-coding RNAs (lincRNAs) analysis with high accuracy and sensitivity. LincRNAs are long non-coding transcripts from the intergenic regions of annotated protein-coding genes. A current study analyzed the lncRNA LOC152742 using quantitative reverse transcriptase-polymerase chain reaction (qRT-PCR) in normal subjects, active TB patients, obsolete TB patients, and individuals affected by BCG (Bacillus Calmette-Guerin) in sputum [103]. Additionally, LOC152742 expression levels were also analyzed in lung epithelial cells and macrophages infected with either H37Ra or H37Rv [103]. The results showed that the specificity of LOC152742 in the sputum and plasma was higher in active TB than in obsolete TB and BCG patients. Also, the expression level of LOC152742 was significantly higher in lung epithelial cells and macrophages infected with either H37Ra or H37Rv than in the uninfected group, which further indicates the potential of LOC152742 to be a novel biomarker for TB.

Another study also pointed out that the lncRNAs, ENST00000354432 and ENST00000427151, in sputum were up-regulated in TB patients [15]. Furthermore, in this study, after a series of experimental data analyses, the authors verified that ENST00000354432 and ENST00000427151 could be potential molecular biomarkers for the rapid diagnosis of TB [15]. These results demonstrate the potential prospect of using lncRNAs as a more sensitive and accurate biomarker for diagnosing TB based on sputum samples.

### 4.6. LncRNAs from Exosomes as Potential Biomarkers in TB

Exosomes are extracellular vesicles with an approximate size of 30–100 nm [127]. The production of exosomes involves the formation of endosomes and the biogenesis of multivesicular bodies (MVBs), which could fuse with the cell membrane and finally be released outside the cell [128]. With the continuous exploration of exosomes in recent years, increasing pieces of evidence have indicated that exosomes released from various cells can act as mediators of information exchange between different cells and are a more precise molecular mechanism involved in the process of intercellular communication (Figure 1G) [129].

It has been found that pulmonary exosomes are derived from a wide range of respiratory cell types, such as structural and immune cells. These exosomes are able to present different amounts of components depending on the disease state with a distinct disease-specific component that may provide new diagnostic biomarkers for a wide range of lung diseases [130]. Numerous studies have shown that exosomal lncRNAs play an important role in different systemic diseases, including cardiovascular [131], neurological [132], and urinary diseases [133]. In recent years, new advances in exosomal lncRNAs have also been made in respiratory diseases. A recent study identified nine dysregulated lncRNAs in the serum exosomes of active TB patients through a comprehensive analysis of the GEO dataset (GSE94907) and the NONCODE database. Compared to healthy controls, the expression of NONHSAT101518.2, NONHSAT067134.2, NONHSAT148822.1, and NONHSAT078957.2 were significantly down-regulated in the plasma exosomes of active TB patients (*p* < 0.0001) [107]. Meanwhile, receiver operating characteristic (ROC) curve analysis showed that the area under the curves (AUCs) were 0.8994 (95% CI 0.8443–0.9545) for NONHSAT078957.2, 0.8725 (95% CI 0.8064–0.9386) for NONHSAT067134.2, 0.9502 (95% CI 0.9153–0.9852) for NONHSAT101518.2, and 0.7080 (95% CI 0.6207–0.7954) for NONHSAT148822.1, respectively [107]. These findings suggest that these four lncRNAs exhibit high sensitivity and specificity in distinguishing patients with active TB from healthy individuals. Additionally, this work also indicated that exosomal lncRNAs might have the potential to be non-invasive diagnostic biomarkers and therapeutic targets for PTB [129].

### 4.7. LncRNAs from Other Samples as Potential Biomarkers in TB

A recent study analyzed serum lncRNA NORAD expression in PTB patients and evaluated its potential clinical significance [108]. These researchers assessed NORAD serum levels using qRT-PCR in 90 PTB patients and 85 healthy individuals. They found that PTB patients exhibited a marked increase in NORAD levels (*p* < 0.001), and that this rise was positively associated with the concentrations of the inflammatory cytokines IL-1β (r = 0.854), TNF-α (r = 0.617), and IL-6 (r = 0.585). These results imply that the irregularity in NORAD expression may correspond to the overproduction of inflammation in PTB. To further explore the diagnostic significance of NORAD in tuberculosis, the researchers then analyzed the receiver operating characteristic curves. They found that when the serum NORAD level was more remarkable than 1.317, the AUC was 0.918, with a sensitivity of 80.0% and a specificity of 89.4%. NORAD significantly differentiated tuberculosis patients from healthy individuals (95% CI of 0.879–0.958, *p* < 0.001). Therefore, NORAD may become a potential clinical diagnostic biomarker for PTB.

Another study discovered that dysbiosis of the gut microbiota can lead to a more severe infection of tuberculosis [109]. This study identified a range of symbiotic bacteria-related lncRNAs, among which the lncRNA CGB was significantly downregulated in mice infected with tuberculosis bacilli with gut microbiota imbalance. Meanwhile, the researchers conducted a comprehensive analysis of the gut microbiota in fecal samples from individuals with active tuberculosis and healthy subjects utilizing 16S rRNA gene sequencing. They found that the gut microbiota in TB patients was characterized by decreased richness and diversity. Bioinformatics analysis was then performed to compare the two RNA expression profiles, and it was found that the expression levels of CGB were significantly lower in TB patients than in healthy individuals. Therefore, the lncRNA CGB could be a potential biomarker for TB.

## 5. Conclusions and Perspectives

Here, we discussed the potential of lncRNAs as novel biomarkers of TB for clinical applications. As more and more researchers have devoted themselves to lncRNA research in recent years, it makes lncRNAs emerging hot molecules in clinical medicine research. However, the current understanding of lncRNAs’ function is only the tip of the iceberg due to the complex and diverse mechanisms of lncRNAs’ action. Given the diversity of lncRNAs types and functions, there are still difficulties in how to distinguish between functional and non-functional non-coding transcripts. To date, there are few bioinformatics tools for lncRNA prediction. Additionally, to elucidate the delicate molecular mechanisms of lncRNAs, more advanced high-resolution imaging techniques and highly sensitive detection technologies are needed. We believe that with the advancement of technology, the functions of lncRNAs and their molecular regulatory mechanisms, as well as their pathological mechanisms in the development of diseases, will be elucidated.

Compared with other biomarkers, lncRNA has its inborn advantages as a novel biomarker for TB. Compared with mRNA, lncRNAs have the advantage of being functional molecules, meaning that their expression level could more accurately reflect the disease status with high expression patterns. However, the current research on lncRNAs is only in its infancy compared to protein-coding sequences and small molecule RNAs, which leaves many gaps in understanding the regulatory mechanisms of lncRNAs. Some important issues remain to be further unexplored, such as whether circulating lncRNAs are stable and representative when detected in body fluids. Therefore, increasing the in-depth exploration and attention to the field of lncRNA research is significant for further research on the mechanism of TB infection and the development of novel diagnostic biomarkers or therapeutic targets for TB.

In TB diagnosis, current efforts are focused on how to accurately diagnose patients with PTB without microbiological evidence of Mtb infection. LncRNA prediction models are expected to largely improve the clinical diagnosis of PTB compared to traditional tests. Currently, novel nomograms incorporating lncRNA features and electronic health record (EHR) data have been proven to be effective in distinguishing clinically diagnosed PTB patients from those with suspected diseases, which are also expected to develop a promising diagnostic approach for PTB patients with negative microbiological evidence [100]. In addition, the higher abundance of lncRNAs compared to protein-coding genes provides a larger window for detecting biomarkers of disease-subtype-specific lncRNAs. The expression of subtype/tissue-specific lncRNAs is critical for the development of new biomarkers and personalized therapies [134,135]. LncRNAs are large and can fold into complex secondary/tertiary structures and scaffolds, which in turn can interact with various proteins, transcriptional regulators, mRNAs, and DNA sequences [136]. And the presence of a large number of regulatory sites of lncRNAs functions provides a broader platform for the development of novel structure-based anti-TB drugs.

Effective screening of TB is a method for treating TB in a timely manner and controlling the disease’s progression. Microarrays, RNA sequencing, PCR-based approaches, in situ hybridization (ISH), and lncRNA knockdown have always been used for detecting lncRNA. However, the aforementioned techniques face obstacles, such as technical constraints, practicability, and long detection times [16]. As discussed above, lncRNAs from the peripheral blood, plasma, tissues, etc., are potential biomarkers for TB. Therefore, the development of a point-of-care device utilizing existing technologies, such as nanotechnology-based tools, is very likely to achieve the goal of the rapid, sensitive, and timely detection of lncRNAs, permitting the real-time, reproducible monitoring of lncRNAs for TB disease risk assessment [137].

The feasibility of lncRNAs as biomarkers is enhanced by the fact that they are stable and monitorable in body fluids and can be assessed using non-invasive procedures [138]. Compared with a single lncRNA as a biomarker for TB diagnosis, combining several kinds of lncRNAs has demonstrated a more potent ability for disease diagnosis [105]. This combination could avoid the errors associated with using only a single lncRNA that might result in misdiagnosis and would allow for the comprehensive detection of samples for a more accurate and sensitive diagnosis of TB. Additionally, it might be more sensitive and accurate to develop diagnostic methods that combine functional lncRNAs with other potential biomarkers for future TB diagnosis, which would benefit from the potential misdiagnosis introduced by the single lncRNA biomarker.

The substantial HIV seroprevalence among individuals with active tuberculosis demonstrates the overlap between HIV and tuberculosis. Due to the higher virulence of Mtb in comparison to other less harmful organisms, TB is frequently an early symptom of HIV infection [139]. To date, most clinical diagnostic methods have inherent limitations for TB/HIV co-infection [140]. It is particularly important to develop new tests to improve diagnostic efficiency and accuracy. Some ncRNAs are considered biomarkers during TB or HIV infection; for example, the miR-889 target can be manipulated for antifungal therapeutic purposes as well as being a candidate biomarker for LTBI [141]. Thus, lncRNAs, as biomarkers of the host immune response, may provide key insights to address this issue [16]. Furthermore, by identifying the key lncRNAs related to HIV-1 susceptibility, replication, and latency, along with understanding their molecular mechanisms of action, it may be possible to design specialized and targeted HIV-1 therapy methods [142].

Compared with the typical pulmonary TB (PTB), intestinal TB (ITB) is more difficult to be diagnosed, even with contemporary medical procedures, due to its unspecific clinical and biochemical features. Currently, a mix of clinical, endoscopic, imaging, and pathologic characteristics is used to diagnose ITB [143]. The microbiologic diagnosis of cutaneous tuberculosis can be challenging as most skin lesions only contain a limited number of mycobacteria that are unable to be detected though staining or culture. In such circumstances, polymerase chain reaction-based nucleic acid amplification assays have potential [144]. Furthermore, previous studies have suggested that most lncRNAs linked with infectious illnesses are aberrantly expressed in diverse tissues and cells with lower lncRNA cellular concentrations but higher tissue specificity than RNAs encoding proteins [10]. This finding indicates that lncRNAs may also be implicated in other common TB diseases, but more research into their mechanisms of action are needed to provide new ideas for their diagnostic and therapeutic application.

Drug resistance is one of the most urgent issues for TB, which has introduced several challenges in drug-resistant TB prevention, diagnosis, and therapy. The rise of multidrug-resistant strains, in particular, as well as the HIV epidemic, have undermined efforts to control TB [145]. As a result, there is an urgent need to identify unique biomarkers, as well as accessible technical methods, for earlier diagnosis of drug-resistant TB. It is worth noting that there is growing evidence indicating that lncRNAs play critical roles in regulating tumorigenesis, invasion, metastasis, and drug resistance [146,147]. In addition, there is growing evidence that lncRNAs are widely expressed and involved in the occurrence and progression of many diseases, including TB [148]. For example, it has been demonstrated that n335659 is differentially expressed in the sera of MDR-TB, DS-TB, and healthy individuals and is thereby suitable for its use as a biomarker for MDR-TB [149]. Notably, mutations in drug-resistant Mtb are often difficult to predict. Thus, more attention should also be paid to exploring the relationships between lncRNAs and drug-resistant TB, which may provide novel insights for the prevention, diagnosis, and therapy of drug-resistant TB.

Although strong potentials have been introduced, there are still a lot of challenges to overcome before the actual use of lncRNAs as clinical diagnostic biomarkers or targets; for example, the specific expression of lncRNAs in TB patients when compared with other diseases and the instability of oligonucleotides. Due to the complicated roles of lncRNAs, many of the lncRNAs changed under the course of Mtb infections have also been revealed to exhibit differences under other microbial infections or other diseased conditions, which means that these altered lncRNAs cannot be used as specific biomarkers for TB. Thus, how to explore the novel lncRNA candidates that are specific for TB remains a challenge. Due to the nonspecific changes of lncRNAs in disease, we believe that combining the novel TB diagnosis strategy using lncRNAs as biomarkers with the current TB diagnosis strategy would be a more sensible choice currently, which could further enhance the accuracy of diagnostic results. Also, our understanding of the interactions between lncRNAs and other genes or proteins is still limited, which dramatically restricts our confidence in developing specific lncRNAs for actual clinical application. Therefore, the successful application of lncRNAs in TB diagnosis requires an unprecedented interdisciplinary approach, including technological advances in molecular biology, immunology, pharmacology, chemistry, and nanotechnology, to overcome the obstacles faced in their clinical application. However, their successful translation still requires further interdisciplinary developments to confirm the exact relationship between the stability, specificity, and other gene or protein interactions of lncRNAs.

In recent years, there has been an increasing number of new tools for the diagnosis of TB, especially multidrug-resistant TB. The WHO has endorsed two types of genotyping: molecular beacon analysis and line probe hybridization analysis [150]. The WHO has approved the use of the molecular beacon assay Xpert MTB/RIF as a common initial diagnostic test for multidrug-resistant TB, which can detect resistance to rifampicin in less than two hours, but the high cost and false-negative rate limits its use [149]. Thus, a combination of changes based on lncRNAs levels and bioinformatics analyses can be used to improve the understanding of the pathomechanisms of drug-resistant TB, leading to the development of more applicable assays.

Our presentation provides new research ideas and insights into the potential clinical application of lncRNAs in TB, especially their roles as biomarkers for diagnostic uses. But we cannot deny that there are still lots of obstacles hindering the diagnostic application of lncRNAs. More experiments and observations are needed to assess the specificity and safety of lncRNAs for the diagnosis and treatment of TB. Although challenging, we believe that the scientific obstruct to the use of lncRNAs in TB diagnosis will eventually be removed due to more promising and creative developments in the fundamental research of lncRNAs. And, as a functional molecule with muscular tissue and cell-type specificity, lncRNAs will play an increasingly important role in developing novel TB diagnostic strategies.

**Strategy and Keywords Employed for Literature Search**

**Keywords**: lncRNAs (long non-coding RNAs) + Tuberculosis + Biomarkers/Diagnosis.

**Databases:** PubMed/MEDLINE, Google Scholar, Web of Science, the National Center for Biotechnology Information, Ensembl, NONCODE, and the National Genomics Data Center.

**Strategy:** Combine each keyword or its synonyms in different database for literature search; apply additional filters or limits, if necessary; scan the titles and abstracts of the retrieved articles for relevance to the review topic; retrieve full texts of relevant articles; and repeat the search periodically to include the most recent publications.

## Figures and Tables

**Table 1:** lncRNAs for potential diagnostic uses in TB.

Type	LncRNA	Biological Property	No. of Patients/Controls	Sample Source	Up/Down-Regulation	AUCs	*p*-Value	Reference
Active TB/Latent TB	AC007128.1	Specifically expressed in TB patients	900/1534	Peripheral blood	Up	_	=0.012	[96]
Active TB/Latent TB	AP001065.3	Its expression profile showed significant differences in active TB, latent TB, and healthy controls.	900/1534	Peripheral blood	Up	_	=0.018	[96]
TB	NEAT1	May act as a transcriptional regulator for many genes, including some implicated in advancing malignancies	106/55	PBMCs	Up	_	<0.01	[97]
PTB	MIR3945HG V1	Based expression in the appendix (RPKM 2.1), lung (RPKM 1.7), and nine other tissues	31/32	PBMCs	Up	0.925	<0.001	[98]
PTB	MIR3945HG V2	Based expression in the appendix (RPKM 2.1), lung (RPKM 1.7), and nine other tissues	31/32	PBMCs	Up	0.956	<0.001	[98]
PTB	n344917	Highly expressed in both lymphocytes and leukocytes	238/298	PBMCs	Down	0.88	<0.001	[99]
PTB	ENST00000497872	Differently expressed in PTB patients	637/1127	PBMCs	Down	0.89	<0.0001	[100]
PTB	n333737	Differently expressed in PTB patients	637/1127	PBMCs	Down	0.89	<0.0001	[100]
PTB	n335265	Differently expressed in PTB patients	637/1127	PBMCs	Up	0.89	<0.05	[100]
PTB	TCONS_00001838	Differently expressed in PTB patients	805/1954	PBMCs	Up	0.86	<0.001	[101]
PTB	n406498	Differently expressed in PTB patients	805/1954	PBMCs	Down	0.86	<0.001	[101]
Active TB	CD244	Regulated by CD244 molecule and biased expression in the spleen (RPKM 7.3), bone marrow (RPKM 3.3), and 12 other tissues	_	PBMCs	Up	_	<0.05	[26]
Untreated TB/cured TB	uc.48+	Could effectively distinguish between the untreated TB group and the cured TB group	50/29	Plasma	Up	0.945	<0.001	[102]
Untreated TB/cured TB	NR_105053	Restricted expression toward testis (RPKM 4.8)	50/29	Plasma	Up	0.945	=0.03	[102]
Active TB/Obsolete TB/BCG patients	LOC152742	High specificity in sputum and plasma (95% CI, 88.62 and 86.21) in active TB patients	48/44	Plasma	up	0.923/0.852/0.874	<0.0001	[103]
Active PTB	ENST00000354432	Have stable nucleic acid splicing sites, a low base replacement rate, a self-deletion rate, a foreign fragment insertion rate, and a space-specific expression mode.	53/106	Plasma	Up	_	<0.05	[15]
Active PTB	ENST00000427151	Ubiquitous expression in fat (RPKM 5.1), endometrium (RPKM 3.8), and 25 other tissues.	53/106	Plasma	Up	_	<0.05	[15]
TB and Latent TB	LINC00870	Regulates Th1/Th2 by the JAK/STAT pathway in MTB-infected PBMCs.	52/25	Plasma and sputum	Up	_	<0.05	[104]
Active TB	NR_038221	Interacts with mRNA and miRNA to form a regulatory network that exerts a significant effect on TB.	114/105	Plasma	Up	0.677	<0.01	[105]
Active TB	NR_003142	Ubiquitous expression in bone marrow (RPKM 9.9), salivary gland (RPKM 6.8), and 23 other tissues	114/105	Plasma	Up	0.657	<0.05	[105]
Active TB	ENST00000570366	Had a positive correlation with CD3E and IL5	114/105	Plasma	Up	0.672	<0.05	[105]
Active TB	ENST00000422183	Had a positive correlation with IL6ST and a negative correlation with TLR6	114/105	Plasma	Down	0.738	<0.001	[105]
Sputum-negative TB	ENST00000429730.1	Strong interactions were found with proteins of ELAV1, TRA2B, PTBP1, SRSF9, and SRS10	11/-	Lung tissue	Up	0.750	=0.024	[106]
Sputum-negative TB	MSTRG.93125.4	Strong interactions were found with proteins of SFPQ, PCBP1, LN28B, SRSF2, and PCBP2	11/-	Lung tissue	Up	0.813	=0.001	[106]
Active PTB/obsolete TB/BCG patients	LOC152742	High specificity in sputum and plasma (95% CI, 88.62 and 86.21) in active TB patients.	48/44	Sputum	Up	0.914/0.836/0.838	<0.0001	[103]
Active PTB	ENST00000354432	Have stable nucleic acid splicing sites, a low base replacement rate, self-deletion rate, foreign fragment insertion rate, and a space-specific expression mode	53/106	Sputum	Up	_	<0.05	[15]
Active PTB	ENST00000427151	Ubiquitous expression in fat (RPKM 5.1), endometrium (RPKM 3.8), and 25 other tissues	53/106	Sputum	Up	_	<0.05	[15]
Active TB	NONHSAT148822.1	High expression in normal-people-blood (FPKM: 5719.16) in exosome expression profile	79/79	Plasma	Down	0.708	<0.0001	[107]
Active TB	NONHSAT078957.2	High expression in normal-people-blood (FPKM: 129.665) in exosome expression profile	79/79	Plasma	Down	0.8994	<0.0001	[107]
Active TB	NONHSAT067134.2	High expression in the HepG2-cell line (FPKM: 20166.5) in the exosome expression profile	79/79	Plasma	Down	0.8725	<0.0001	[107]
Active TB	NONHSAT101518.2	High expression in normal-people-blood (FPKM:1266.23) in the exosome expression profile	79/79	Plasma	Down	0.9502	<0.0001	[107]
PTB	NORAD	Involvement of Mtb-infected macrophage activity and inflammation by targeting miR-618	90/85	Serum	Up	0.918	<0.001	[108]
Active TB	lncRNA-CGB	Interacted with EZH2 and down-regulation of H3K27 tri-methylation epigenetic programming contributes to increasing the expression of IFN-γ	56/49	Fecal	Down	_	<0.01	[109]

**Table 2:** Advantages and challenges of lncRNAs as potential biomarkers for TB.

lncRNAs as Biomarkers to Diagnose TB		Present Diagnostic Methods
Advantages	Challenges	
1. Has the potential to identify cases of PTB with negative microbiological evidence of *Mycobacterium tuberculosis* [100]. 2. May distinguish between untreated TB patients and cured TB subjects [16]. 3. Can provide effective diagnosis of early TB [105]. 4. High sensitivity and specificity [107]. 5. Can be detected easily and rapidly from a number of human body fluids using non-invasive procedures [110].	1. LncRNA is unstable and easily degraded, and extraction and preservation in clinical applications remains to be explored. 2. In these studies mentioned, “specificity” pertains to comparing samples from healthy volunteers and patients with a particular disease [111]. In this particular context, “specificity” does not refer to the ability to differentiate one disease from another. Therefore, whether lncRNAs are significantly differentially expressed in other diseases needs further investigation.	1. Interferon-gamma release assays (IGRAs). 2. Tuberculin skin tests (TSTs). 3. Acid-fast bacilli (AFB) smear. 4. Nucleic acid amplification test (NAAT). 5. Rapid molecular drug susceptibility testing of respiratory specimens. 6. Mycobacterial culture. 7. Flexible bronchoscopy. 8. Histologic examination. 9. Adenosine deaminase measurement.

## Data Availability

Not applicable.
